# Neddylation inhibitor MLN4924 has anti‐HBV activity via modulating the ERK‐HNF1α‐C/EBPα‐HNF4α axis

**DOI:** 10.1111/jcmm.16137

**Published:** 2020-12-02

**Authors:** Mingjie Xie, Huiting Guo, Guohua Lou, Jiping Yao, Yanning Liu, Yi Sun, Zhenggang Yang, Min Zheng

**Affiliations:** ^1^ The State Key Laboratory for Diagnosis and Treatment of Infectious Diseases The First Affiliated Hospital Zhejiang University Hangzhou China; ^2^ Collaborative Innovation Center for Diagnosis and Treatment of Infectious Diseases Hangzhou China; ^3^ Cancer Institute of the Second Affiliated Hospital Institute of Translational Medicine Zhejiang University School of Medicine Hangzhou China; ^4^ Division of Radiation and Cancer Biology Department of Radiation Oncology University of Michigan Ann Arbor MI USA

**Keywords:** HBV, MAPK, MLN4924, neddylation, transcription factors

## Abstract

Hepatitis B virus (HBV) infection is a major public health problem. The high levels of HBV DNA and HBsAg are positively associated with the development of secondary liver diseases, including hepatocellular carcinoma (HCC). Current treatment with nucleos(t)ide analogues mainly reduces viral DNA, but has minimal, if any, inhibitory effect on the viral antigen. Although IFN reduces both HBV DNA and HBsAg, the serious associated side effects limit its use in clinic. Thus, there is an urgent demanding for novel anti‐HBV therapy. In our study, viral parameters were determined in the supernatant of HepG2.2.15 cells, HBV‐expressing Huh7 and HepG2 cells which transfected with HBV plasmids and in the serum of HBV mouse models with hydrodynamic injection of pAAV‐HBV1.2 plasmid. RT‐qPCR and Southern blot were performed to detect 35kb mRNA and cccDNA. RT‐qPCR, Luciferase assay and Western blot were used to determine anti‐HBV effects of MLN4924 and the underlying mechanisms. We found that treatment with MLN4924, the first‐in‐class neddylation inhibitor currently in several phase II clinical trials for anti‐cancer application, effectively suppressed production of HBV DNA, HBsAg, 3.5kb HBV RNA as well as cccDNA. Mechanistically, MLN4924 blocks cullin neddylation and activates ERK to suppress the expression of several transcription factors required for HBV replication, including HNF1α, C/EBPα and HNF4α, leading to an effective blockage in the production of cccDNA and HBV antigen. Our study revealed that neddylation inhibitor MLN4924 has impressive anti‐HBV activity by inhibiting HBV replication, thus providing sound rationale for future MLN4924 clinical trial as a novel anti‐HBV therapy.

## 1 INTRODUCTION

HBV infection is one of the major public health problem with 364 million people being chronically infected by this distinct pathogen worldwide.^1^, ^2^ About 25% of population with chronically infection of HBV in childhood develop serious liver diseases, such as hepatitis, cirrhosis and even HCC.^3^, ^4^ Nearly 1 million persons die from cirrhosis and HCC secondary to HBV infection each year.^5^, ^6^ Current treatment strategy for chronic Hepatitis B (CHB) is to improve life quality and extend survival by slowing the deteriorative process to hepatitis, cirrhosis and HCC. As circulation hallmarks of CHB, high blood levels of viral load (HBV DNA) and HBsAg are responsible for liver cirrhosis and HCC.^7^, ^8^ Currently, the standard nucleos(t)ide analogues therapy only decreases HBV DNA and has minimal, if any, inhibitory effect on HBsAg. Although Interferon inhibits both viral DNA and antigen, the side effects associated limit its use in clinic.^1^, ^9^, ^10^ Therefore, potential new anti‐viral strategies that effectively reduce both HBV DNA and HBsAg levels are highly desirable.

Neddylation, one type of post‐translational modifications of a given protein, is catalysed by three sequential enzymatic reactions. These enzymes are E1 NEDD8‐activating enzyme (NAE), E2 neddylation conjugating enzymes and E3 neddylation ligase. In mammalian cells, there is only one E1, containing a heterodimer of regulatory subunit, NAE1/APPBP1, and a catalytic subunit, UBA3/NAEβ; two E2s, UBE2M and UBE2F; and little over 10 E3s, along with limited number of neddylation substrates.^11^ A well‐characterized physiological substrate of neddylation is a family of Cullin‐RING Ligase (CRL), which is the largest family of the E3 ubiquitin ligase, and responsible for ubiquitination of 20% cellular proteins for degradation by proteasome system.^12^, ^13^ CRL is a multi‐component E3 ligase, consisting of a scaffold protein, cullin (with 8 family members, Cullins 1‐3, 4A, 4B, 5, 7 and 9), an adapt protein (such as S‐phase kinase‐associated protein 1(SKP1)), substrate‐recognizing subunit (eg an F‐box protein), and a RING protein family member, ROC1/RBX1 or ROC2/RBX2/SAG.^14^, ^15^, ^16^, ^17^, ^18^ Activity of CRLs requires a) the RING component, ROC1 or SAG, which binds to a ubiquitin‐loaded E2, and b) cullin neddylation, which prevents inhibitory binding of CAND1.^13^, ^19^

MLN4924, also known as pevonedistat, is a small‐molecule inhibitor of E1 NAE, discovered 10 years ago.^12^ By inhibiting E1, MLN4924 blocks the entire neddylation modification and inactivates all family members of CRLs.^19^ As CRLs are frequently overexpressed in many types of human cancers,^19^ MLN4924 has shown impressive anti‐cancer activity in extensive preclinical settings against a variety of human cancer cells by inducing growth arrest, apoptosis, autophagy and senescence.^11^ Currently, MLN4924 is in several phases II clinical trials for the treatment of haematological malignancies and solid tumours, mainly in combination with conventional chemotherapies.^11^, ^12^, ^20^, ^21^, ^22^ Interestingly, in addition to anti‐cancer application, MLN4924 has been shown to have broad activity against various viruses,^23^ including HIV,^24^, ^25^, ^26^, ^27^, ^28^, ^29^, ^30^ influenza A virus,^31^ and most recently, HCV,^27^ mainly through inactivation of CRLs to cause accumulation of anti‐viral proteins. Two groups reported that MLN4924 has anti‐HBV activity with mechanism involving restoration of Smc5/6 protein levels to suppress viral replication.^32^, ^33^ However, the detailed mechanism underlying the function of the MLN4924 in silencing HBV replication remains elusive.

In this study, we systematically investigated anti‐HBV activity of MLN4924 and the underlying mechanism. We found that in both cell culture and animal models, HBV infection activates cullin neddylation, and MLN4924 effectively suppressed HBV replication and HBsAg production. Our mechanistic study revealed that MLN4924 activates ERK to block expression of transcription factors HNF1α, C/EBPα and HNF4α, which is required for viral replication. Our results suggest the potential use of MLN4924 as an alternative therapeutic strategy for HBV treatment.

## 2 MATERIAL AND METHODS

### 2.1 Cell lines

The human hepatoma cell lines HepG2 and Huh7 cells were purchased from American Type Culture Collection and were cultured in Dulbecco's modified Eagle medium (DMEM) with 10% foetal bovine serum(FBS) (Gibco, Carlsbad, Calif, USA).^34^ Cell lines HepG2.2.15 which express HBV persistently were purchased from the Chinese Center For Type Culture Collection (CCTCC, Wuhan, China) and were maintained in DMEM with 400 μg/ml G418 as well as10% FBS.^35^ HepAD38 cells were kindly provided by Min Chen from Chongqing Medical University and were maintained in DMEM with 12% FBS. All the cells were kept in 37°C with 5% CO_2_. Cell viability was assessed by the Cell Counting Kit‐8(CCK‐8)(Dojindo Laboratories).

### 2.2 Animals

C57BL/6 mice (male, 6‐8 weeks old) in animal tests were purchased from Shanghai Laboratory Animal Center (Shanghai, China). Ten micrograms of pAAV‐HBV1.2 plasmid DNA in a volume of PBS equivalent to 8% of mouse weight were injected via tail vein in 5s according to the previous method.^36^ The mice were divided into MLN4924 group and vehicle (10% hydroxypropyl‐beta‐cyclodextrin (HP‐β‐CD)) group 5 days after injection.Mice then injected MLN4924 (60 mg/kg bodyweight) or vehicle (10%HP‐β‐CD) by Intraperitoneal at indicated times.^31^ The serum was extracted for viral DNA and antigen tests at the indicated times. The mice's liver tissues were kept in Tissue Optimum Cutting Temperature (OCT)‐freeze Medium for immunohistochemistry analysis. All mice were maintained under specific pathogen‐free conditions in the Laboratory Animal Center of Zhejiang University. The experiments were conducted in accordance with the Guide for the Care and Use of Laboratory Animals. The experimental schedule has been approved by the Ethics Review and Scientific Investigation Board of The First Affiliated Hospital, Zhejiang University.

### 2.3 Compounds

MLN4924 (HY‐10484, MedChemExpress USA) was purchased from MedChem Express and dissolved in dimethyl sulphoxide (DMSO) (Sigma‐Aldrich, St. Louis, MO, USA) to make a 10mM to 100 mM solution and stored at −80℃. In animal experiments, the drug was dissolved in 10% HP‐β‐CD (Sangon Biotech Inc, Shanghai, China). U0126 (HY‐12031, MedChemExpress USA) was purchased from MedChemExpress and dissolved in DMSO (Sigma‐Aldrich, St. Louis, MO, USA) to generate a 1 mM solution and stored at −40℃. Tenofovir (HY‐13782A, MedChemExpress USA) was purchased from MedChemExpress and dissolved in DMSO (Sigma‐Aldrich, St. Louis, MO, USA) to generate a 1 mM solution and stored at −40℃. Tetracycline (HY‐B0474, MedChemExpress USA) was purchased from MedChemExpress and dissolved in DMSO (Sigma‐Aldrich, St. Louis, MO, USA) to generate a 100mg/ml solution and stored at −40℃.

### 2.4 Plasmids and antibodies

The plasmid encoding pAAV‐HBV1.2 was kindly provided by Pei‐Jer Chen from the Department of Internal Medicine, National Taiwan University Hospital, National Taiwan University College of Medicine. HBV promoters including X promoter (XP), core promoter (CP), PreS1 promoter (preS1P), PreS2 promoter (preS2P) luciferase report vectors (pGL3‐Xp, pGL3‐S1p, pGL3‐S2p and pGL3‐Cp) were created in our laboratory according to previous studies.^37^ pHBV1.37 plasmid was generated in our laboratory according to previous studies.^37^ The antibodies used were listed: anti‐p‐ERK (4370S, Cell Signaling Technology, USA), anti‐ERK (4695S, Cell Signaling Technology, USA), anti‐HNF1α (89670S,Cell Signaling Technology,USA), anti‐HNF4a(3113S, Cell Signaling Technology,USA), anti‐C/EBPa (8178S, Cell Signaling Technology, USA), anti‐PPARa (ab3484,abcam,USA), anti‐NEDD8 (ab81264, abcam, USA), anti‐GAPDH (2118S, Cell Signaling Technology, USA), anti‐Actin (A1015,DAWEN BIOTECH,CHINA) Normal Rabbit IgG (WD‐GAR007, DAWEN BIOTECH,CHINA),Normal Mouse IgG (GAM007,MULTI SCIENCES,CHINA).

### 2.5 Transfection

Lipofectamine 3000 (L3000015, Invitrogen, USA) was used to transfect plasmid. The procedure was carried out according to the instructions.

### 2.6 Luciferase assay

96‐well plates which contain 1.0 × 10^4^ HepG2 cells or Huh7cells per well were transiently transfected with 120ng HBV reporter plasmid and 15 ng pRL‐TK plasmid. Add MLN4924 to the drug‐proceeded group 6 hours after transfection. The luciferase activities were measured by GloMax microplate luminometer (Promega, USA) using Dual‐Glo® Luciferase Assay System kit (E2920, Promega, USA) according to the instructions.

### 2.7 Total RNA extraction and real‐time (RT) qPCR

Total RNA samples were extracted via RNAiso Plus (9109, TaKaRa Bio, Japan). PrimeScript™ RT reagent Kit with gDNA Eraser (Perfect Real Time) (RR047A, TAKARA Bio, Japan) was used for reverse transcription. ABI 7900HT Fast Instrument (Applied Biosystems, USA) was applied for quantitative PCR using SYBR® Premix Ex Taq™ II (RR820A, TAKARA Bio, Japan). Primers used in the tests were obtained from Sangon Co.Ltd (Shanghai, China).

### 2.8 Immunohistochemistry

Mice Liver tissues were collected and kept in OCT. Immunohistochemical was used to detect intrahepatic HBcAg by staining anti‐HBc(ZA‐0121,ZSGB‐BIO,CHINA); intrahepatic NEDD8‐Cullins by staining anti‐NEDD8(ab81264, abcam, USA) according to previous methods.^38^

### 2.9 HBV DNA and antigen detection

HBV DNA in cell supernatants and serum were detected by the Fluorescence Quantitative PCR Detection Kit for Hepatitis B Virus DNA (ACON Biotech Co. Ltd, Hangzhou, China). Viral antigen in cell supernatants and serum including HBsAg and HBeAg were measured by Abbott i2000SR (Abbott Diagnostics, Abbott Park, IL, USA) using Architect HBsAg and HBeAg Reagent kits (Abbott Diagnostics, Abbott Park, IL, USA).

### 2.10 HBV cccDNA detection

Huh7 transfected with pHBV1.37 were lysed in lysis buffer within proteinase K (QIAGEN), total DNA was extracted according to a standard phenol‐chloroform extraction protocol. The total DNA was digested with plasmid‐safe adenosine triphosphate (ATP)‐dependent deoxyribonuclease DNase (PSAD) (Epicentre Technologies) for 8 h at 37°C. DNase was inactivated by incubating the reactions for 30 min at 70°C. The digested DNA was used for quantification of HBV cccDNA within HBV cccDNA specific primers: 5'TGCACTTCGCTTCACCT3’ (forward) and 5'AGGGGCATTTGGTGGTC3’ (reverse).The real‐time PCR was performed using the SYBR Premix Ex Taq on ABI 7900 Fast Real‐Time PCR System as the following reaction procedure: 95°C for 5 min then 45 cycles of 95°C for 30 s, 62°C for 25 s and 72°C for 30 s.

### 2.11 Alanine aminotransferase and aspartate aminotransferase measurement

Serum alanine aminotransferase (ALT) and aspartate aminotransferase (AST) was measured with HESKA Dri‐Chem 4000 (HESKA; slides from Fujifilm, Tokyo, Japan).

### 2.12 Western blot

Proteins extracted from cells and tissues were boiled for 10 min at 100 ℃ and resolved on 4‐20% gradient SDS‐PAGE gel (Genescript) subsequently transferred to PVDF membranes. The membranes were blocked in 1XTBS‐T containing 5%BSA for 40 minutes and incubated in primary following corresponding secondary antibodies. The bands were visualized by ChemiScope 3300 Mini equipment (CLINX, Shanghai, China) using EZ‐ECL Kit HRP (Biological industries, Israel).

### 2.13 Southern blot

The viral DNAs used in southern blot were extracted from cells according to methods: Dissolve each 60mm cell culture plate cells with 1.5ml TE buffer (10:10) and 0.1ml 10% sodium dodecyl sulphate (SDS), then incubate at room temperature for 40 minutes. Transfer the cell lysate to a clean 15 mL centrifuge tube, then add 0.4 mL 5 M NaCl, and gently flip the tube. The tube was then incubated at 4°C for 24 hours. Centrifuge at 15 000 × g for 30 minutes at 4°C. Transfer the supernatant to a new 15 mL centrifuge tube. Add the same amount of phenol to the supernatant and mix it thoroughly by shaking for 10 s. Centrifuge at 4000 × g for 10 minutes at 4°C and transfer the aqueous phase to a new 15 mL middle tube. Add two volumes of 100% ethanol, invert the test tube several times. Incubate tube overnight at room temperature to precipitate DNA. On the second day, centrifuge at 4000 × g for 30 minutes at 4°C and discard the supernatant. Add an equal amount of 75% ethanol to wash the DNA particles. Centrifuge at 4000 × g for 15 minutes at 4°C. Discard the supernatant. Let the pellets air dry for about 10 minutes at room temperature. Dissolve DNA particles in 20μL TE buffer (10:1).^39^ Hirt method was used to detect cccDNA, as described previously.^40^, ^41^ The isolated DNA samples were separated on 0.9%agarose gel then transferred to nylon membrane following hybridized with HBV‐specific probe according to the instructions.^40^The primers for HBV‐specific probe: 5’AATTCCACAACCTTTCACCAAACTC3’(Forward);5’CACTGCATGGCCTGAGGATGAGT’(Reverse).

### 2.14 Statistical analysis

Results were analysed by GraphPad Prism v7.0a (GraphPad Software, Inc, SanDiego, USA). Data were presented as mean ± SEM. Results of significance were using Student t test and *P* < .05 was considered statistically significant.

## 3 RESULTS

### 3.1 HBV activates cullin neddylation

**Figure 1:**
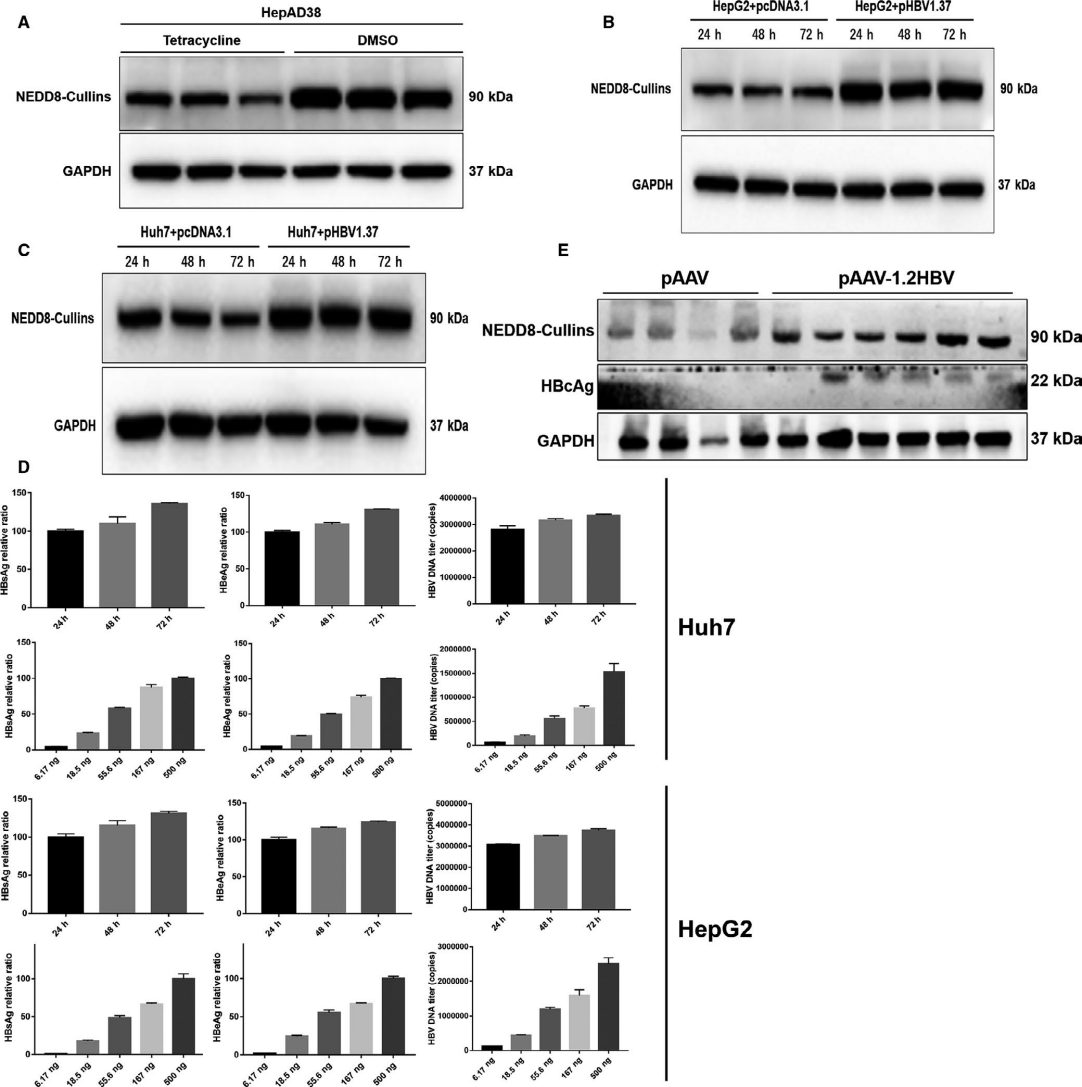
HBV activates cullin neddylation in hepatoma cells and liver tissues. (A) HepAD38 cells were cultured in 6 well plates and treated with tetracycline (1μg/μL) or not until confluent. The cell lysates were harvested for Western blotting for NEDD8‐Cullins. (B) HepG2 cells and (C) Huh7 cells were transfected with pHBV1.37 plasmids. Cells were collected at indicated times after transfection and subjected to Western blotting using indicated Abs.(D) HepG2 cells and Huh7 cells were transfected with pHBV1.37 plasmids in a time‐dependent manner(the concentration of plasmids were 1μg/mL) as well as with different dose of plasmids(culture mediums were collected for HBV detection after 48h transfection) separately. Culture mediums were collected for RT‐qPCR to determine the HBV DNA levels and subjected to ELISA to measure the levels of HBsAg and HBeAg. (E) 10 μg of pAAV‐HBV1.2 plasmid, along with empty vector control, were injected into C57BL/6 mice through the tail vein. The mice livers were collected from 4 controls and 6 experimental mice 4 days after injection, followed by Western blotting with indicated antibodies. Cells were then harvested for protein lysate preparation, followed by Western blotting with indicated Abs

Previous studies have shown that MLN4924 has anti‐viral activity in various virus models by inactivating CRLs (Cullin‐RING ligases). To determine potential involvement of CRLs in HBV expression, we used HBV stable‐expression cell line HepAD38. As tetracycline can completely control HBV replication in HepAD38 cells and the viral replication can be greatly inhibited in the presence of tetracycline,^42^ we treated HepAD38 cells with tetracycline or not. We found that the levels of NEDD8‐Cullins are higher in tetracycline‐absent group (Figure 1A). We also transiently transfected HBV‐expressing plasmid pHBV1.37 into hepatoma HepG2 and Huh7 cells and detected again elevated levels of neddylated cullins in both lines (Figure 1B&C). We show the HBV replication in a time‐dependent manner as well as with different dose of transfection of HBV plasmid in the meanwhile (Figure 1D). More importantly, using in vivo mouse model, we detected increased levels of neddylated cullins in liver tissues derived from mice 4 days after tail‐vein injection of pAAV‐HBV1.2 plasmids (Figure 1E). Taken together, our results showed that HBV viral plasmids elevated the levels of neddylated cullins both in vitro and in vivo models, suggesting a potential involvement of CRLs in HBV infection.

### 3.2 MLN4924 inhibits the HBV replication and antigen production in vitro

**Figure 2:**
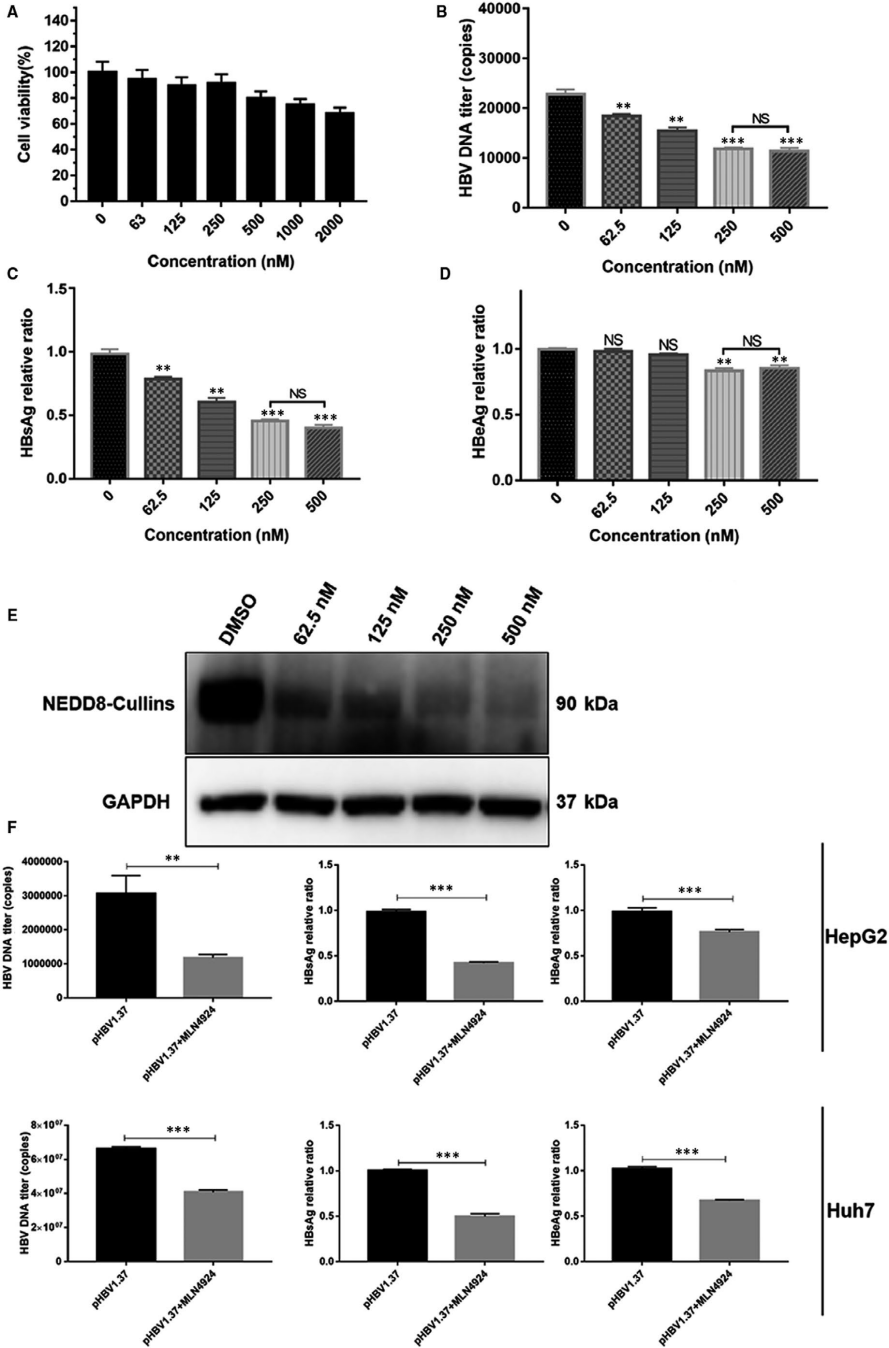
MLN4924 suppresses HBV in HBV‐expressing liver cells. (A) Cytotoxicity of MLN4924. HepG2.2.15 cells were treated with MLN4924 at the indicated concentrations for 48h, followed by CCK8 growth assay. (B‐D) Effect of MLN4924 on HBV replication: HepG2.2.15 cells were treated with MLN4924 at indicated concentration for 48h. Culture mediums were collected for RT‐qPCR to determine the HBV DNA levels (B) and subjected to ELISA to measure the levels of HBsAg (C) and HBeAg (D). (E) HepG2.2.15 cells were treated with MLN4924 at the indicated concentrations along with DMSO control for 48 h. The cell lysates were then harvested for Western blotting for NEDD8‐Cullins. HepG2 cells and Huh7 cells (F) were transiently transfected with pHBV1.37. Cells were treated 6 h later with MLN4924 for 48 h. Culture mediums were collected for RT‐qPCR to determine the HBV DNA levels and subjected to ELISA to measure the levels of HBsAg and HBeAg.** *P* < .01, *** *P* < .001, NS: no significance

We then investigated whether MLN4924, a potent inhibitor of neddylation E1‐activating enzyme,^12^ can inhibit HBV replication in in vitro cell culture setting. We first tested MLN4924 cytotoxicity and found that in HepG2.2.15 cells, MLN4924 at 500 nM caused less than 20% of growth inhibition (Figure 2A). We found that MLN4924 significantly reduced the levels of secreted HBV DNA and HBsAg, as well as HBeAg (to a less extent) in culture supernatants in a dose‐dependent manner (Figure 2 B‐D). Using these doses, we showed the concentration of NEDD8‐Cullins (Figure 2E). We further confirmed the inhibitory effect of MLN4924 on HepG2 (Figure 2F) and Huh7 (Figure 2F) cells after transiently transfection of plasmid encoding pHBV1.37.Tenofovir Disoproxil Fumarate (TDF) has been widely used as first‐line agents for the treatment of infection of HBV in clinic.^43^ It was shown that TDF significantly inhibited HBV DNA (Figure S1A), but it had no obvious effect on the production of HBsAg and HBeAg (Figure S1B‐C). Collectively, MLN4924 showed significant anti‐HBV activity in cell culture settings. Given that anti‐viral effect of MLN4924 is similar between concentrations of 250nM and 500nM, we used MLN4924 at 250nM for the rest of study.

### 3.3 MLN4924 inhibits HBV particles and the levels of HBV antigen in vivo

**Figure 3:**
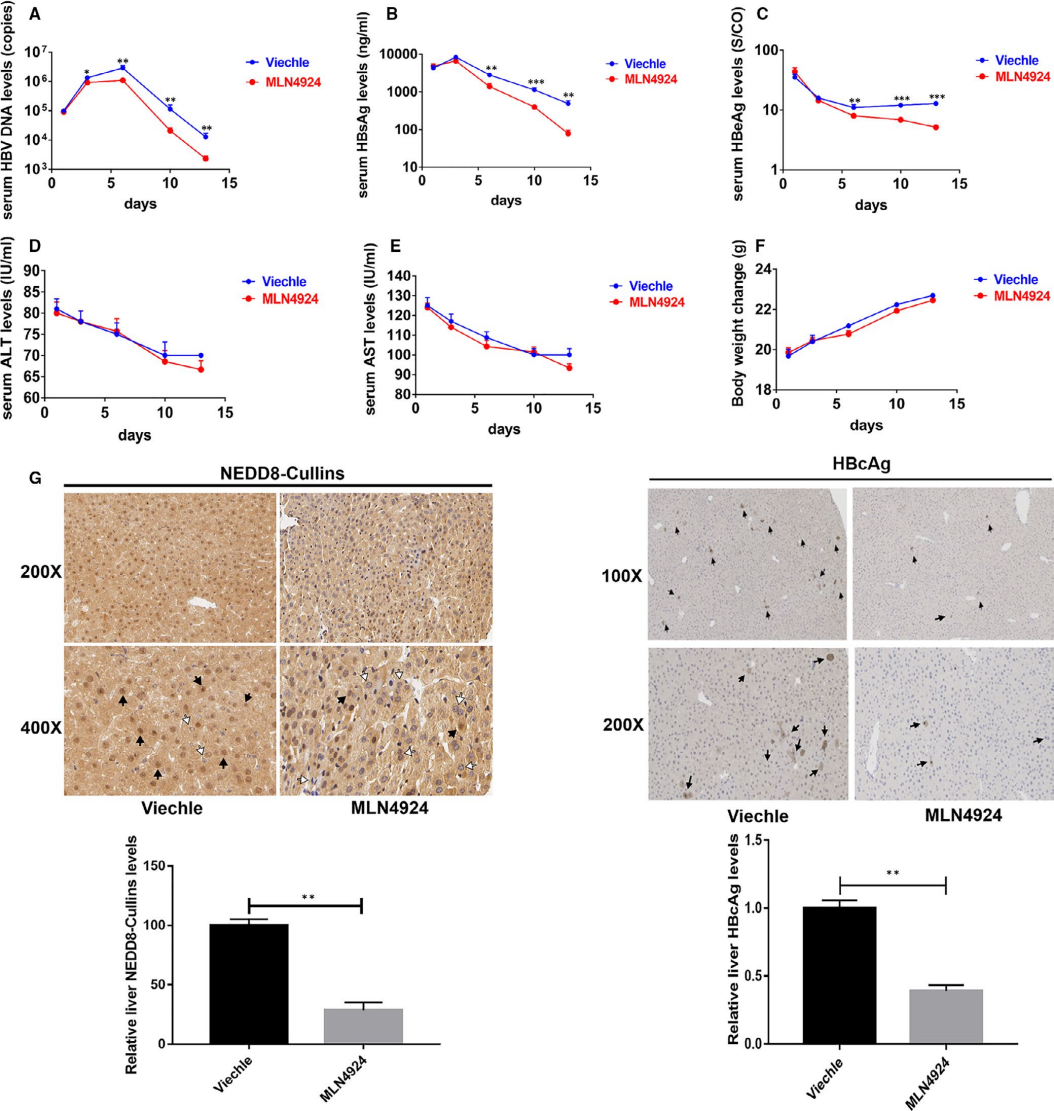
MLN4924 exhibits anti‐HBV activity in HBV‐mice models. C57BL/6 mice were hydrodynamically injected with 10 μg of pAAV‐HBV1.2 plasmids through tail vein. The mice were subsequently injected i.p. with 60 mg/kg of MLN4924 at day 0, 1, 3, 5, 7 and 9 after injection. Serum samples were collected at indicated time‐points. The HBV DNA replicative intermediates in the serum were evaluated by RT‐qPCR (A), whereas the levels of HBsAg (B) and HBeAg (C) were measured by ELISA, along with measurement of ALT and AST (D,E), and bodyweight (F). Mice livers were collected at 13‐day after virus injection, fixed, sectioned for immunohistochemistry staining with anti‐NEDD8 and anti‐HBcAg Ab separately. Shown are representative areas (Positive cells were indicated with solid arrows) (G). * *P* < .05, ** *P* < .01, *** *P* < .001

We further explored whether MLN4924 has anti‐HBV activity in vivo. C57BL/6 mice were injected via the tail vein with 10 μg of pAAV‐HBV1.2 plasmid.^36^ The mice were subsequently injected i.p. with 60 mg/kg of MLN4924 at day 0, 1, 3, 5, 7 and 9 post‐injection. Serums were collected at various time‐points after MLN4924 dosing. Indeed, MLN4924 significantly reduced the levels of serum HBV DNA (Figure 3A), HBsAg (Figure 3B) and HBeAg (Figure 3C) without affecting liver function nor growth, as evidenced by similar serum levels of ALT and AST (Figure 3D&E), and bodyweight (Figure 3F) between treated and control mice. Immunohistochemical analysis showed that the NEDD8‐Cullins (Mainly nucleus expressing) and HBcAg expression were decreased in the liver of MLN4924‐treated mice (Figure 3G). These results indicated that MLN4924 indeed has anti‐HBV activity at non‐toxic dose in vivo.

### 3.4 MLN4924 inhibits production of HBV 3.5kb RNA and cccDNA and blocks HBV promoter activity

**Figure 4:**
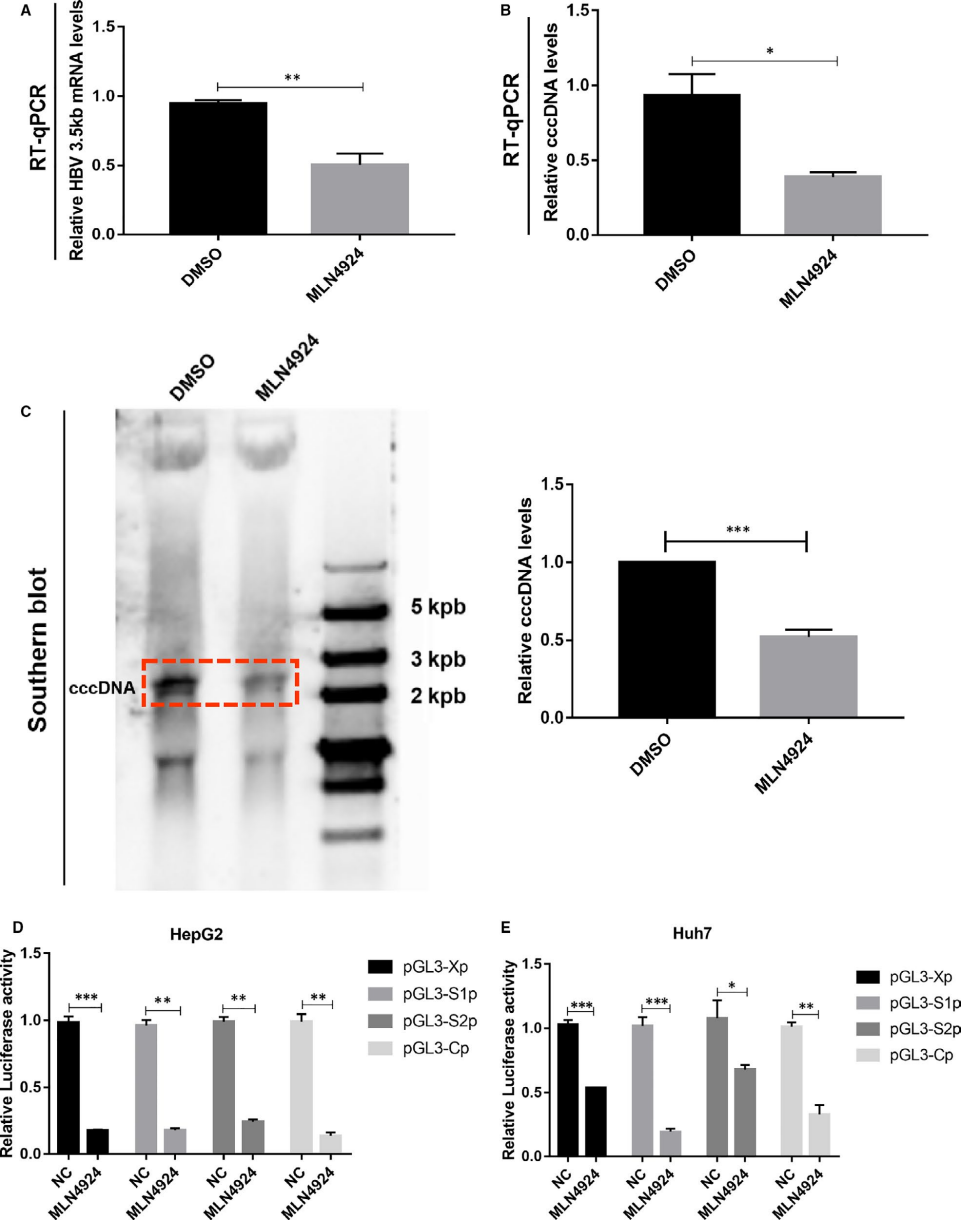
MLN4924 inhibits HBV transcription and replication. Total RNA was isolated from HepG2.2.15 cells with or without treatment of MLN4924 and subjected to RT‐qPCR analysis to measure the levels of HBV 3.5kb RNA (A). (B‐C) Cells were harvested and the levels of cccDNA were detected by RT‐qPCR (B) and Southern blot (C). Activities of HBV promoters were assessed by luciferase‐based reported assay in HepG2 (D) and Huh7 (E) cells treated with MLN4924 or vehicle control for 48 h, as indicated. Shown are mean ± SEM from three independent experiments. *P < .05, ** *P* < .01, *** *P* < .001

To explore how MLN4924 suppressed HBV, we used RT‐qPCR to measure the levels of HBV 3.5 kb RNA in HepG2.2.15 cells (with stable HBV expression) and found a significant reduction upon MLN4924 treatment (Figure 4A). Significantly, MLN4924 treatment also reduced the levels of the cccDNA in Huh7 cells transfected with pHBV 1.37 plasmid, as measured by RT‐qPCR (Figure 4B) and Southern blot (Figure 4C). Finally, we determined the effects of MLN4924 on HBV promoter activities, using luciferase‐based reporters driven by XP, preS1P, preS2P and CP which represent respectively the promoters that drive the expression of the genes encoding HBV X protein, HBV large surface protein, HBV middle and small surface protein, HBV core protein in HepG2 (Figure 4D) and Huh7 (Figure 4E) cells. Again, MLN4924 significantly inhibited the activities of these HBV promoters. Taken together, HBV replication and transcription were significantly inhibited by MLN4924.

### 3.5 MLN4924 inhibits expression of several transcription factors required for HBV replication

**Figure 5:**
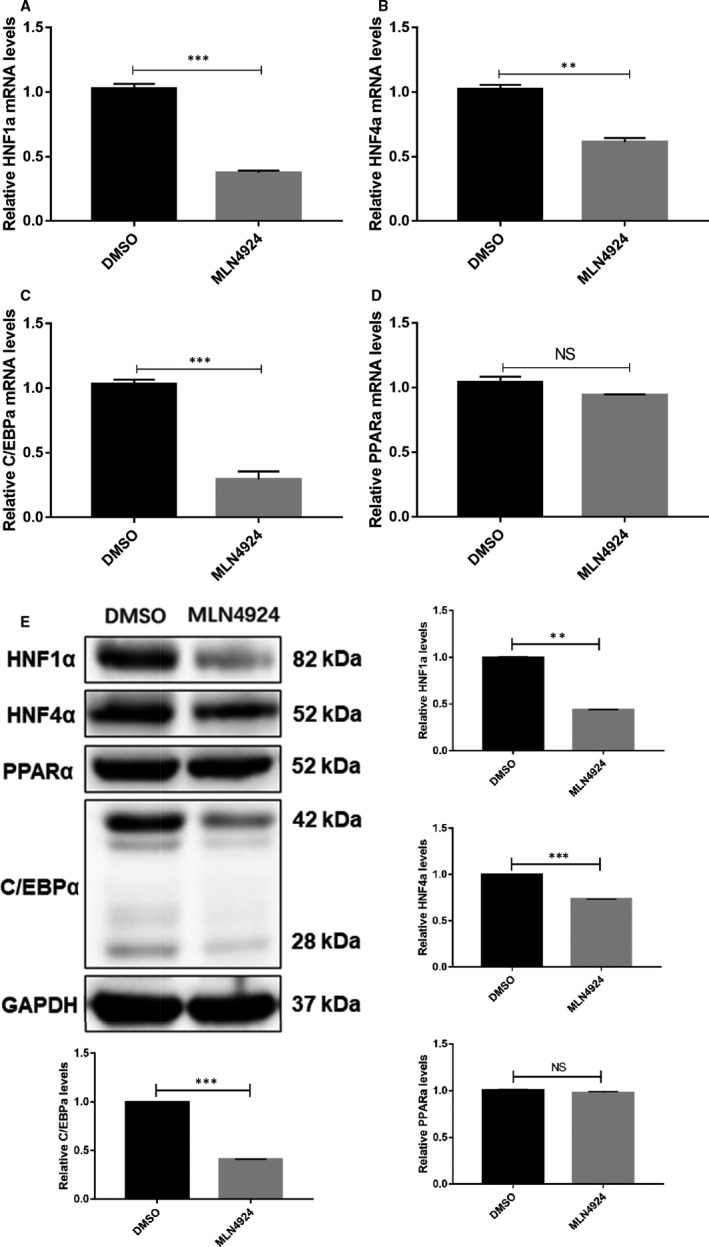
MLN4924 suppressed the expression of HNF1α, HNF4α and C/EBPα, required for HBV transcription. HepG2.2.15 cells were treated with 250 nM MLN4924, along with DMSO control for 48 h. Cells were then harvested for total RNA isolation or protein lysate preparation, followed by RT‐PCR analysis for indicated transcription factor (A‐D) and Western blotting with indicated Abs (E). Shown are mean ± SEM from three independent experiments ** *P* < .01, *** *P* < .001, NS, no significance

Several transcription factors are required in activation of HBV promoters, including peroxisome proliferator‐activated receptor (PPARα), C/EBPα, HNF4α and HNF1α.^44^, ^45^ We next determined whether MLN4924 has any effect on the expressions of these transcription factors using both real‐time PCR and Western blotting in HepG2.2.15 cells. The results clearly showed that MLN4924 down‐regulated the expression of HNF1α, C/EBPα and HNF4α, but not PPARα at both mRNA (Figure 5A‐D) and protein levels (Figure 5 E), providing a molecular mechanism by which MLN4924 suppresses HBV transcription and replication.

### 3.6 MLN4924 anti‐HBV activity is mediated by activation of MAPK signal

**Figure 6:**
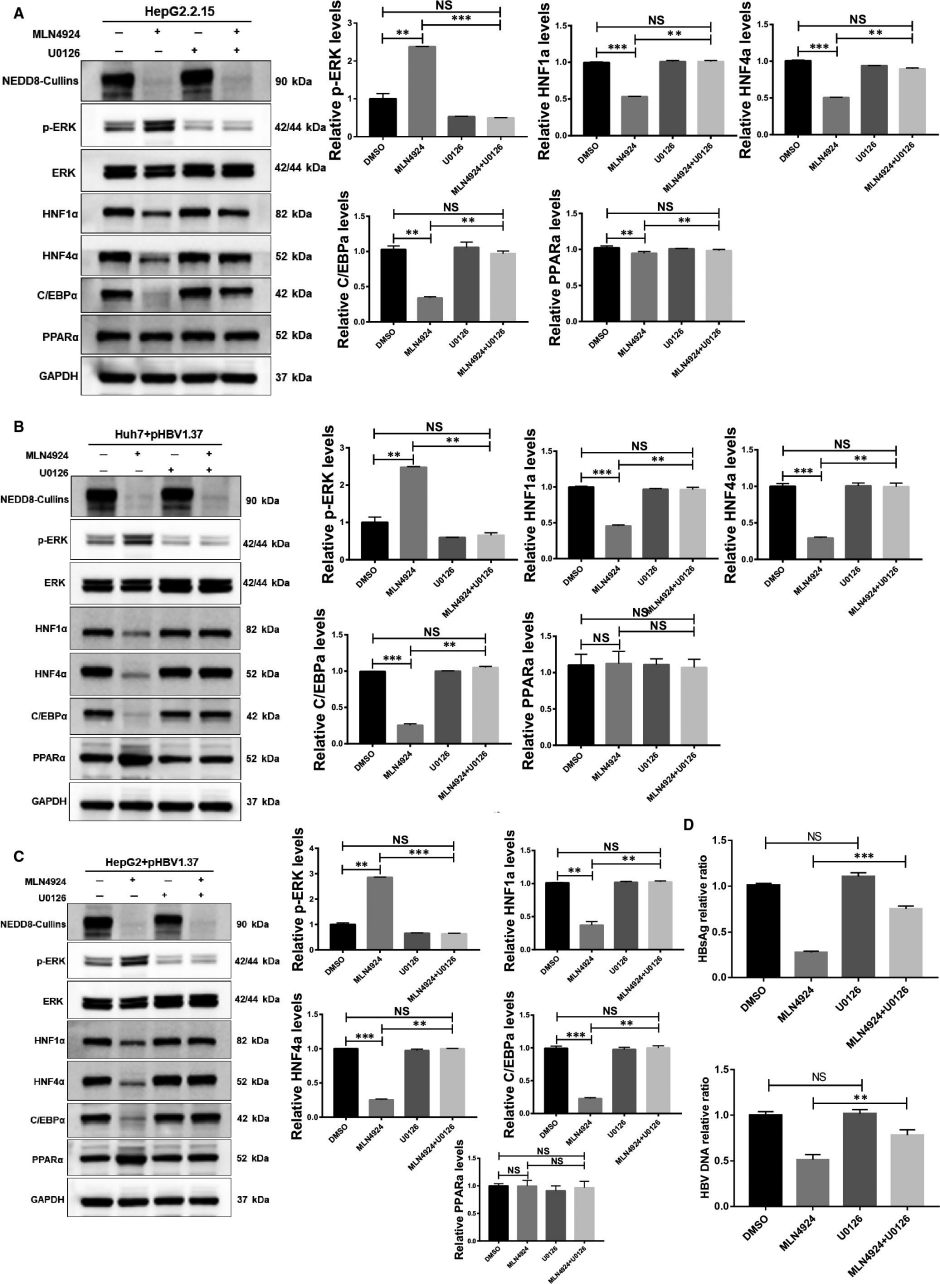
pERK plays a critical role in mediating MLN4924 anti‐HBV activity. The HepG2.2.15 cells were treated with MLN4924 (250 nM), U0126 (10 uM), alone or in combination for 48 h, followed by Western blotting with indicated Ab (A). HepG2 (B) or Huh7 (C) cells were transfected with pHBV1.37 plasmid, treated with MLN4924 or U0126 alone or in combination and followed by Western blot analysis using indicated Ab. Supernatants collected in HepG2.2.15 (D) were subjected to RT‐qPCR and ELSA measurement of HBV DNA and HBsAg levels separately. Densitometry quantification of Western blots was conducted using Image J. *P < .05, ** *P* < .01, *** *P* < .001, NS: no significance

We have previously shown that MLN4924 triggers EGFR dimerization to activate ERK (pERK),^46^ whereas pERK was reported to suppress HBV ^46^, ^47^, ^48^ and negatively regulates HNF1α, C/EBPα and HNF4α.^49^ We, therefore, determined whether the pERK was involved in MLN4924‐induced HBV suppression. We first confirmed that MLN4924 treatment indeed activated ERK in HepG2.2.15 cells, as evidenced by increased phosphor‐ERK (pERK), which was blocked by MEK inhibitor, U0126 (Figure 6A). We then used all three cellular HBV‐expressing models and found that MLN4924 reduced the levels of transcription factors HNF1α, C/EBPα and HNF4α, which can be rescued by MEK inhibitor U0126, while U0126 had no effect if acting alone (Figure 6A‐C). Finally, MLN4924‐mediated anti‐HBV activity can be largely rescued by U0126 (Figure 6D), strongly suggests a causal role of pERK in mediating MLN4924 suppression of HBV.

## 4 DISCUSSION

Previous studies have shown that MLN4924 has broad anti‐virus activity, mainly by inactivation of CRLs. For example, MLN4924 anti‐HIV activity was mediated by inhibiting CRL5‐induced degradation of APOBEC3G ^24^ or CRL4‐induced degradation of SAMHD1.^25^ The anti‐influenza virus activity of MLN4924 was mediated by blocking NFκB nuclear translocation to reduce secretion of pro‐inflammatory cytokines,^31^ whereas anti‐HCV activity was achieved by impairing the function of VPR.^27^ In the case of HBV, two studies reported that X protein of hepatitis B virus promotes degradation of SMC5/6 via CRL4 to enhance HBV replication.^32^, ^33^ A most recent study showed that MLN4924 has anti‐HBV activity by restoring SMC5/6 levels via inactivating CRL4.^50^ Whether the anti‐virus activity of MLN4924 can also be mediated by a mechanism other than CRLs inactivation is previously unknown.

In this study, with a portion of it reported last year in an international symposium,^51^ we used both HBV‐infected in vitro cell culture and in vivo mouse models to test anti‐HBV activity of MLN4924. We first found that HBV infection activated cullin neddylation in all three cellular models, which is effectively inhibited by MLN4924 (Figure 1). We further showed that MLN4924 at non‐toxic doses inhibited HBV DNA titre and the levels of HBV antigens HBsAg and HBeAg both in vitro and in vivo in dose and time‐dependent manner (Figure 2 and Figure 3). This is achieved by MLN4924‐induced abrogation of activities of a number of HBV promoters, leading to reduced levels of 3.5 kb HBV RNA and cccDNA (Figure 4).

What is the possible mechanism by which MLN4924 down‐regulated the promoter activity of HBV? We turned our attention to four liver‐enriched transcription factors, HNF1α, HNF4α, C/EBPα and PPARα, which are not only important regulators for liver metabolic homeostasis,^45^ but also shown to bind HBV promoter/enhancer elements to activate HBV transcription.^52^, ^53^, ^54^ Specifically, HNF1α enhances viral transcription by activating a) HBV preS1P activity via binding to its enhancer/promoter,^55^, ^56^ and b) HBV CP activity via combining HBV Enh II B2 region.^57^ While HNF4α overexpression increases activities of preS1P, preS2P and CP,^48^, ^58^, ^59^ C/EBPα binds and activates the HBV Enh II, CP and preS2P.^52^ We found that MLN4924 effectively reduced the levels of HNF1α, HNF4α and C/EBPα without affecting PPARα (Figure 5), providing a molecular explanation of how MLN4924 suppresses viral promoter activity.

We further pursued how MLN4924 reduces the protein levels of these cellular transcription factors. The effect is unlikely due to direct inhibition of CRLs, since CRLs inactivation would cause an increase, not decrease of substrates. A previous study has showed that in human hepatoma cells, activation of MAPK signal down‐regulates HNF‐4 expression and completely inhibits C/EBPα expression with compromised recruitment of HNF‐3β and HNF‐1α to the HNF‐4 enhancer, and RNA polymerase II to the proximal HNF‐4 promoter,^49^ indicating MPAK signal is a negative regulator of these liver transcription factors. Furthermore, RAS‐MAPK activation by external stimuli has previously shown to suppress HBV replication in both Huh7 and HepG2 cells.^60^

Is there any connection between MLN4924 and MAPK activation? Indeed, we recently found that in addition to blocking cullin neddylation as a potent NAE inhibitor, MLN4924 also activates EGFR and downstream AKT1 and ERK1/2 signals by triggering EGFR dimerization in lung, breast and colon cancer cells.^46^ Here, we showed that in all three EBV‐infected liver cell models, MLN4924 activates MAPK signal leading to increased ERK1/2 phosphorylation, and ERK1/2 activation inhibits protein levels of HNF1α, HNF4α and C/EBPα (Figure 6). More importantly, inactivation of pERK1/2 by a MEK inhibitor U0126 rescued MLN4924 effects, as evidenced by restoring the levels of these three transcription factors, and abrogating inhibition in the production of HBV DNA and HBsAg (Figure 6). Taken together, we conclude that MAPK activation plays a causal role in anti‐HBV activity of MLN4924.

**Figure 7:**
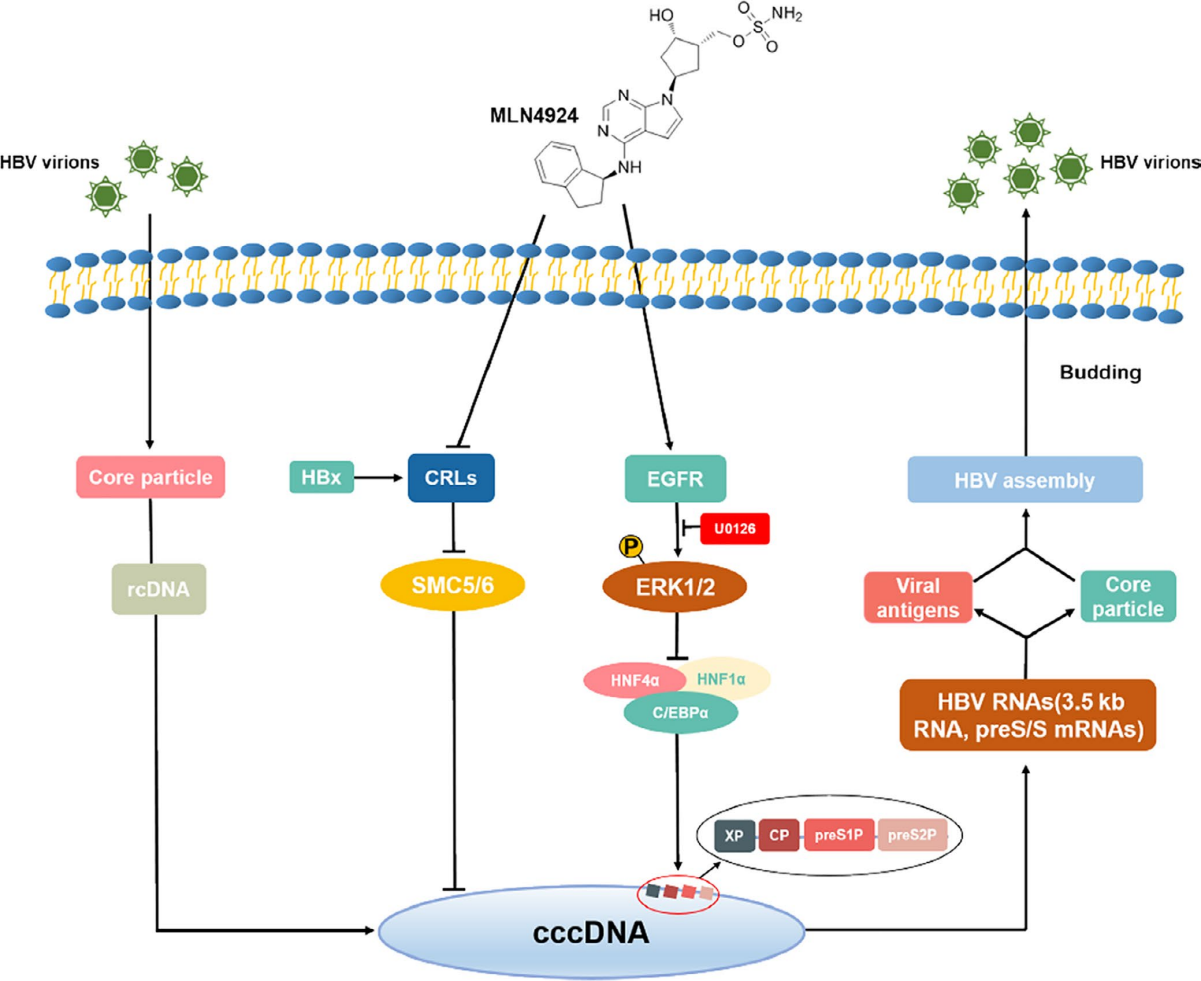
MLN4924 anti‐HBV model. MLN4924 has two distinct mechanisms of action. On one hand, MLN4924 inactivates CRLs to restore the levels of SMC5/6 to suppress cccDNA,^50^ and on the other hand, it activates ERK via EGFR signals to down‐regulate the expression of HNF1α, HNF4α and C/EBPα. Together, MLN4924 inhibits activities of various HBV promoters, leading to reduction of HBV RNA, HBsAg and rcDNA and finally virion production

In summary, we made two novel observations in this study: First, HBV infection in all three cellular models activates CRLs by enhancing cullin neddylation. The underlying mechanism is unknown at the present time, but it is certainly an interesting subject for future investigation; and second, anti‐HBV activity of MLN4924 can also be achieved by activation of MAPK signal, which suppresses few transcription factors that drive HBV transcription. Our study, along with a recent publication,^50^ supports the following model for MLN4924 anti‐HBV activity. MLN4924, on one hand, inactivates CRL4 to restore the levels of SMC5/6 to block cccDNA synthesis, and on the other hand, activates MAPK signals to suppress transcriptional activity of HFN1α, HFN4α and C/EPBα, leading to inhibition of viral promoters of S1p, S2p, Cp and Xp to reduce the levels of 3.5 kb HBV RNA, and eventually reduced HBV rcDNA and HBsAg and HBcAg (Figure 7). It has been reported that CHB patients with the high levels of HBV DNA and HBsAg are more frequently progressed to HCC.^8^ Thus, simultaneous inhibition of viral DNA and antigens will be an ideal approach for anti‐HBV therapy. MLN4924 is a highly selective small‐molecule inhibitor of NEDD8 and can block the entire neddylation modification cascade effectively.^61^ In addition to well‐characterized anti‐neddylation activity, recent studies showed that MLN4924 has several neddylation‐independent activities including the ERK activation we found in this work.^46^, ^61^ So the anti‐HBV activation of MLN4924 may involve neddylation inhibition as well as other mechanisms. Therefore, the degree of NEDD8 inhibition after medication may not be parallel to the inhibition efficiency of HBV and the detailed mechanisms need further research. Taken together, our study showed that MLN4924 is an effective anti‐HBV agent by blocking both viral DNA and antigen, thus providing a sound rational for future clinical trial of this new application.

## CONFLICT OF INTEREST

The authors confirm that there are no conflicts of interest.

## AUTHOR’S CONTRIBUTIONS

Min Zheng, Zhenggang Yang, Yi Sun designed the research; Mingjie Xie, Guohua Lou, Huiting Guo, Jiping Yao performed the experiments and analysed the data, along with Min Zheng and Yi Sun; Mingjie Xie drafted the manuscript; Min Zheng, Zhenggang Yang, Yanning Liu revised and Yi Sun finalized the manuscript.

## Supporting information

Fig S1

## References

[1] Polaris OC . Global prevalence, treatment, and prevention of hepatitis B virus infection in 2016: a modelling study. The lancet Gastroenterol Hepatol. 2018;3(6):383‐403. 29599078 10.1016/S2468-1253(18)30056-6

[2] Fiel MI . Pathology of chronic hepatitis B and chronic hepatitis C. Clin Liver Dis. 2010;14(4):555‐575. 21055682 10.1016/j.cld.2010.07.001

[3] Nannini P , Sokal EM . Hepatitis B: changing epidemiology and interventions. Arch Dis Child. 2017;102(7):676‐680. 27986700 10.1136/archdischild-2016-312043

[4] Chisari FV , Isogawa M , Wieland SF . Pathogenesis of hepatitis B virus infection. Pathologie‐biologie. 2010;58(4):258‐266. 20116937 10.1016/j.patbio.2009.11.001 PMC2888709

[5] McMahon BJ . Natural history of chronic hepatitis B. Clin Liver Dis. 2010;14(3):381‐396. 20638020 10.1016/j.cld.2010.05.007

[6] Hamborsky JKA , Wolfe S . Centers for Disease Control and Prevention. Epidemiology and Prevention of Vaccine‐preventable diseases. Washington DC: Public Health Foundation; 2015;149‐174.

[7] Lok AS , Zoulim F , Dusheiko G , Ghany MG . Hepatitis B cure: From discovery to regulatory approval. J Hepatol. 2017;67(4):847‐861. 28778687 10.1016/j.jhep.2017.05.008

[8] Mueller H , Wildum S , Luangsay S , et al. A novel orally available small molecule that inhibits hepatitis B virus expression. J Hepatol. 2018;68(3):412‐420. 29079285 10.1016/j.jhep.2017.10.014

[9] Pant K , Yadav AK , Gupta P , Rathore AS , Nayak B , Venugopal SK . Humic acid inhibits HBV‐induced autophagosome formation and induces apoptosis in HBV‐transfected Hep G2 cells. Sci Rep. 2016;6. 10.1038/srep34496 PMC5052648 27708347

[10] Stasi C , Silvestri C , Voller F . Emerging Trends in Epidemiology of Hepatitis B Virus Infection. J Clin Translat Hepatol. 2017;5(3):272‐276. 10.14218/JCTH.2017.00010 PMC5606973 28936408

[11] Zhou L , Zhang W , Sun Y , Jia L . Protein neddylation and its alterations in human cancers for targeted therapy. Cell Signal. 2018;44:92‐102. 29331584 10.1016/j.cellsig.2018.01.009 PMC5829022

[12] Soucy TA , Smith PG , Milhollen MA , et al. An inhibitor of NEDD8‐activating enzyme as a new approach to treat cancer. Nature. 2009;458(7239):732‐U67. 19360080 10.1038/nature07884

[13] Zhao Y , Morgan MA , Sun Y . Targeting neddylation pathways to inactivate Cullin‐RING ligases for anti‐cancer therapy. Antioxid Redox Signal. 2014;21(17):2383‐400. 24410571 10.1089/ars.2013.5795 PMC4241876

[14] Duan H , Wang Y , Aviram M , et al. SAG, a novel zinc RING finger protein that protects cells from apoptosis induced by redox agents. Mol Cell Biol. 1999;19:3145‐55. 10082581 10.1128/mcb.19.4.3145 PMC84108

[15] Kamura T , Koepp DM , Conrad MN , et al. Rbx1, a component of the VHL tumor suppressor complex and SCF ubiquitin ligase. Science. 1999;284:657‐61. 10213691 10.1126/science.284.5414.657

[16] Ohta T , Michel JJ , Schottelius AJ , Xiong Y . ROC1, a homolog of APC11, represents a family of cullin partners with an associated ubiquitin ligase activity. Mol Cell. 1999;3:535‐41. 10230407 10.1016/s1097-2765(00)80482-7

[17] Swaroop M , Wang Y , Miller P , et al. Yeast homolog of human SAG/ROC2/Rbx2/Hrt2 is essential for cell growth, but not for germination: Chip profiling implicates its role in cell cycle regulation. Oncogene. 2000;19:2855‐66. 10851089 10.1038/sj.onc.1203635

[18] Sun Y , Tan M , Duan H , Swaroop M . SAG/ROC/Rbx/Hrt, a zinc RING finger gene family: molecular cloning, biochemical properties, and biological functions. Antioxid Redox Signal. 2001;3(4):635‐50. 11554450 10.1089/15230860152542989

[19] Zhao Y , Sun Y . Cullin‐RING ligases as attractive anti‐cancer targets. Curr Pharm Des. 2013;19(18):3215‐25. 23151137 10.2174/13816128113199990300 PMC4034125

[20] Swords RT , Erba HP , DeAngelo DJ , et al. Pevonedistat (MLN4924), a First‐in‐Class NEDD8‐activating enzyme inhibitor, in patients with acute myeloid leukaemia and myelodysplastic syndromes: a phase 1 study. Br J Haematol. 2015;169(4):534‐43. 25733005 10.1111/bjh.13323

[21] Pevonedistat With Azacitidine Versus Azacitidine Alone in Treating Patients With Relapsed or Refractory Acute Myeloid Leukemia(Phase 2). National Cancer Institute (NCI) NCT03745352 NCT03745352 (PHII‐169).

[22] A Randomized Phase II Trial of MLN4924 (Pevonedistat) With Azacitidine Versus Azacitidine in Adult Relapsed or Refractory Acute Myeloid Leukemia(Phase 2). National Cancer Institute (NCI) NCT03745352. (NCT03745352):PHII‐169.

[23] Le‐Trilling VTK , Megger DA , Katschinski B , et al. Broad and potent antiviral activity of the NAE inhibitor MLN4924. Sci Rep. 2016;6:19977. 26829401 10.1038/srep19977 PMC4734293

[24] Stanley DJ , Bartholomeeusen K , Crosby DC , et al. Inhibition of a NEDD8 cascade restores restriction of HIV by APOBEC3G. PLoS Pathog. 2012;8(12):e1003085. 23300442 10.1371/journal.ppat.1003085 PMC3531493

[25] Hofmann H , Norton TD , Schultz ML , Polsky SB , Sunseri N , Landau NR . Inhibition of CUL4A Neddylation causes a reversible block to SAMHD1‐mediated restriction of HIV‐1. J Virol. 2013;87(21):11741‐50. 23986575 10.1128/JVI.02002-13 PMC3807335

[26] Tokarev A , Stoneham C , Lewinski MK , et al. Pharmacologic inhibition of Nedd8 activation enzyme exposes CD4‐induced epitopes within Env on Cells Expressing HIV‐1. J Virol. 2015;90(5):2486‐502. 26676780 10.1128/JVI.02736-15 PMC4810708

[27] Yan Y , Huang F , Yuan T , Sun B , Yang R . HIV‐1 Vpr increases HCV replication through VprBP in cell culture. Virus Res. 2016;223:153‐60. 27460548 10.1016/j.virusres.2016.07.007

[28] Nekorchuk MD , Sharifi HJ , Furuya AK , Jellinger R , de Noronha CM . HIV relies on neddylation for ubiquitin ligase‐mediated functions. Retrovirology. 2013;10:138. 24245672 10.1186/1742-4690-10-138 PMC3842660

[29] DePaula‐Silva AB , Cassiday PA , Chumley J , et al. Determinants for degradation of SAMHD1, Mus81 and induction of G2 arrest in HIV‐1 Vpr and SIVagm Vpr. Virology. 2015;477:10‐7. 25618414 10.1016/j.virol.2014.12.040 PMC4455942

[30] Ramirez PW , DePaula‐Silva AB , Szaniawski M , Barker E , Bosque A , Planelles V . HIV‐1 Vpu utilizes both cullin‐RING ligase (CRL) dependent and independent mechanisms to downmodulate host proteins. Retrovirology. 2015;12:65. 26215564 10.1186/s12977-015-0192-2 PMC4517359

[31] Sun H , Yao W , Wang K , Qian Y , Chen H , Jung YS . Inhibition of neddylation pathway represses influenza virus replication and pro‐inflammatory responses. Virology. 2018;514:230‐9. 29248752 10.1016/j.virol.2017.11.004

[32] Decorsière A , Mueller H , van Breugel PC , et al. Hepatitis B virus X protein identifies the Smc5/6 complex as a host restriction factor. Nature. 2016;531(7594):386‐9. 26983541 10.1038/nature17170

[33] Murphy C , Xu Y , Li F , et al. Hepatitis B virus X protein promotes degradation of SMC5/6 to enhance HBV replication. Cell Rep. 2016;16(11):2846‐54. 27626656 10.1016/j.celrep.2016.08.026 PMC5078993

[34] Nakabayashi H , Taketa K , Miyano K , Yamane T , Sato J . Growth of human hepatoma cells lines with differentiated functions in chemically defined medium. Can Res. 1982;42(9):3858‐63. 6286115

[35] Sells MA , Chen ML , Acs G . Production of hepatitis B virus particles in HepG2 cells transfected with cloned hepatitis B virus DNA. Proc Natl Acad Sci. 1987;84:1005‐9. 3029758 10.1073/pnas.84.4.1005 PMC304350

[36] Huang LR , Wu HL , Chen PJ , Chen DS . An immunocompetent mouse model for the tolerance of human chronic hepatitis B virus infection. Proc Natl Acad Sci U S A. 2006;103(47):17862‐7. 17095599 10.1073/pnas.0608578103 PMC1635544

[37] Guidotti LG , Matzke B , Schaller H , Chisari FV . High‐level hepatitis B virus replication in transgenic mice. J Virol. 1995;69(10):6158‐69. 7666518 10.1128/jvi.69.10.6158-6169.1995 PMC189513

[38] Yu J , Huang W‐L , Xu Q‐G , et al. Overactivated neddylation pathway in human hepatocellular carcinoma. Cancer Med. 2018;7(7):3363‐3372. 10.1002/cam4.1578 PMC6051160 29846044

[39] Ko C , Shin YC , Park WJ , Kim S , Kim J , Ryu WS . Residues Arg703, Asp777, and Arg781 of the RNase H domain of hepatitis B virus polymerase are critical for viral DNA synthesis. J Virol. 2014;88(1):154‐63. 24131721 10.1128/JVI.01916-13 PMC3911735

[40] Guo HJD , Zhou T , Cuconati A , Block TM , Guo JT . Characterization of the intracellular deproteinized relaxed circular DNA of hepatitis Bvirus: an intermediate of covalently closed circular DNA formation. J Virol. 2007;81:12472‐84. 17804499 10.1128/JVI.01123-07 PMC2169032

[41] Yan H , Zhong GC , Xu GW , et al. Sodium taurocholate cotransporting polypeptide is a functional receptor for human hepatitis B and D virus. Elife. 2012;1:e00049. 23150796 10.7554/eLife.00049 PMC3485615

[42] Ladner SK , Otto MJ , Barker CS , et al. Inducible expression of human hepatitis B virus (HBV) in stably transfected hepatoblastoma cells: a novel system for screening potential inhibitors of HBV replication. Antimicrob Agents Chemother. 1997;41(8):1715‐20. 9257747 10.1128/aac.41.8.1715 PMC163991

[43] Delaney WET , Ray AS , Yang H , et al. Intracellular metabolism and in vitro activity of tenofovir against hepatitis B virus. Antimicrob Agents Chemother. 2006;50(7):2471‐7. 16801428 10.1128/AAC.00138-06 PMC1489769

[44] Xia Y , Cheng X , Li Y , Valdez K , Chen W , Liang TJ . Hepatitis B virus deregulates the cell cycle to promote viral replication and a premalignant phenotype. J Virol. 2018;92(19):e00722‐18. 30021897 10.1128/JVI.00722-18 PMC6146796

[45] Chong CL , Chen ML , Wu YC , et al. Dynamics of HBV cccDNA expression and transcription in different cell growth phase. J Biomed Sci. 2011;18:96. 22208719 10.1186/1423-0127-18-96 PMC3262020

[46] Zhou X , Tan M , Nyati MK , Zhao Y , Wang G , Sun Y . Blockage of neddylation modification stimulates tumor sphere formation in vitro and stem cell differentiation and wound healing in vivo. Proc Natl Acad Sci U S A. 2016;113(21):E2935‐44. 27162365 10.1073/pnas.1522367113 PMC4889367

[47] Wu L , Wang W , Zhang X , Zhao X , Yu G . Anti‐HBV activity and mechanism of marine‐derived polyguluronate sulfate (PGS) in vitro. Carbohyd Polym. 2016;143:139‐48. 10.1016/j.carbpol.2016.01.065 27083353

[48] Zhao Z , Hong W , Zeng Z , et al. Mucroporin‐M1 inhibits hepatitis B virus replication by activating the mitogen‐activated protein kinase (MAPK) pathway and down‐regulating HNF4alpha in vitro and in vivo. J Biol Chem. 2012;287(36):30181‐90. 22791717 10.1074/jbc.M112.370312 PMC3436272

[49] Hatzis P , Kyrmizi I , Talianidis L . Mitogen‐activated protein kinase‐mediated disruption of enhancer‐promoter communication inhibits hepatocyte nuclear factor 4 alpha expression. Mol Cell Biol. 2006;26(19):7017‐29. 16980607 10.1128/MCB.00297-06 PMC1592892

[50] Sekiba K , Otsuka M , Ohno M , et al. Pevonedistat, a first‐in‐class NEDD8‐activating enzyme inhibitor, is a potent inhibitor of hepatitis B virus. Hepatology. 2018;69(5):1903–1915. 10.1002/hep.30491 30586159

[51] Xie MJ , Yang F , Yang ZG , Shui LY , Zheng M . Targeting Neddylation, a Potential Strategy in Anti‐HBV Therapy. Hepatology. 2018;68:235a‐6a.

[52] Quasdorff M , Protzer U . Control of hepatitis B virus at the level of transcription. J Viral Hepatitis. 2010;17(8):527‐36. 10.1111/j.1365-2893.2010.01315.x 20546497

[53] Pan Y , Ke Z , Ye H , et al. Saikosaponin C exerts anti‐HBV effects by attenuating HNF1alpha and HNF4alpha expression to suppress HBV pgRNA synthesis. Inflamm Res. 2019;68(12):1025‐34. 31531682 10.1007/s00011-019-01284-2 PMC7079752

[54] Shu Shi ML , Xi J , Liu H , et al. Sex‐determining region Y box 4 (SOX4) suppresses Hepatitis B virus replication by inhibiting hepatocyte nuclear factor 4α expression. Antivir Res. 2020;176:104745. 32084507 10.1016/j.antiviral.2020.104745

[55] Raney AK , Easton AJ , Milich DR , McLachlan A . Promoter‐specific transactivation of hepatitis B virus transcription by a glutamine‐ and proline‐rich domain of hepatocyte nuclear factor 1. J Virol. 1991;65(11):5774‐81. 1656070 10.1128/jvi.65.11.5774-5781.1991 PMC250238

[56] Zhou DX , Yen TSB . The ubiquitous transcription factor Oct‐1 and the liver‐specific factor Hnf‐1 are both required to activate transcription of a hepatitis‐B virus promoter. Mol Cell Biol. 1991;11(3):1353‐9. 1996097 10.1128/mcb.11.3.1353-1359.1991 PMC369406

[57] Wang ML , Wu X , Wang YZ . HNFl is critical for the liver‐specific function of HBV enhancer II. Virology. 1998;149:99‐108. 10.1016/s0923-2516(98)80085-x 9602504

[58] Zi‐Yu Wang Y‐QL , Guo Z‐W , Zhou X‐H , Mu‐Dan LU , Xue T‐C , Gao BO . ERK1/2‐HNF4α axis is involved in epigallocatechin‐3‐gallate inhibition of HBV replication. Acta Pharmacol Sin. 2020;41(2):278‐85. 31554961 10.1038/s41401-019-0302-0 PMC7468327

[59] Lijie Li YL . Zhiqi Xiong, Wangqin Shu, Yuanyuan Yang, Zhiwei Guo, Bo Gao FoxO4 inhibits HBV core promoter activity through ERK‐mediated downregulation of HNF4α. Antivir Res. 2019;170:104568. 31351930 10.1016/j.antiviral.2019.104568

[60] Zheng YY , Li J , Johnson DL , Ou JH . Regulation of hepatitis B virus replication by the Ras‐mitogen‐activated protein kinase signaling pathway. J Virol. 2003;77(14):7707‐12. 12829809 10.1128/JVI.77.14.7707-7712.2003 PMC161924

[61] Mao H , Sun Y . Neddylation‐independent activities of MLN4924. Adv Exp Med Biol. 2020;1217:363‐72. 31898238 10.1007/978-981-15-1025-0_21

