# Supplementary figures and images for: Neddylation inhibitor MLN4924 has anti‐HBV activity via modulating the ERK‐HNF1α‐C/EBPα‐HNF4α axis
Source: J Cell Mol Med. 2020 Dec 2;25(2):840–54. doi: 10.1111/jcmm.16137 (PMC7812279; doi:10.1111/jcmm.16137)

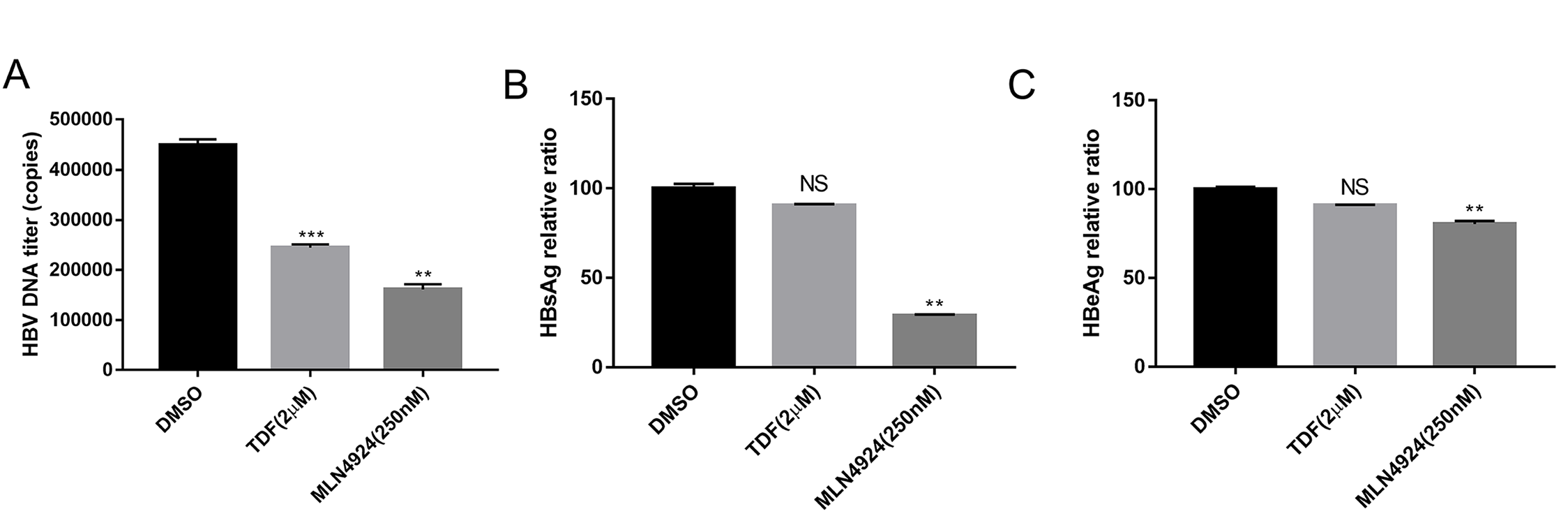

Supplement: Supplementary file 1 — Fig S1 [file JCMM-25-840-s001.tif]
